# Selecting a sample size for studies with repeated measures

**DOI:** 10.1186/1471-2288-13-100

**Published:** 2013-07-31

**Authors:** Yi Guo, Henrietta L Logan, Deborah H Glueck, Keith E Muller

**Affiliations:** 1 Department of Health Outcomes and Policy, College of Medicine, University of Florida, Gainesville, FL, USA; 2 Southeast Center for Research to Reduce Disparities in Oral Health, University of Florida, Gainesville, FL, USA; 3 Department of Community Dentistry and Behavioral Science, College of Dentistry, University of Florida, Gainesville, FL, USA; 4 Department of Biostatistics and Informatics, Colorado School of Public Health, University of Colorado Denver, Aurora, CO, USA

**Keywords:** Sample size selection, Repeated measures, Interaction

## Abstract

Many researchers favor repeated measures designs because they allow the detection of within-person change over time and typically have higher statistical power than cross-sectional designs. However, the plethora of inputs needed for repeated measures designs can make sample size selection, a critical step in designing a successful study, difficult. Using a dental pain study as a driving example, we provide guidance for selecting an appropriate sample size for testing a time by treatment interaction for studies with repeated measures. We describe how to (1) gather the required inputs for the sample size calculation, (2) choose appropriate software to perform the calculation, and (3) address practical considerations such as missing data, multiple aims, and continuous covariates.

## Correspondence

Selecting an appropriate sample size is a crucial step in designing a successful study. A study with an insufficient sample size may not have sufficient statistical power to detect meaningful effects and may produce unreliable answers to important research questions. On the other hand, a study with an excessive sample size wastes resources and may unnecessarily expose study participants to potential harm. Choosing the right sample size increases the chance of detecting an effect, and ensures that the study is both ethical and cost-effective.

Repeated measures designs are widely used because they have advantages over cross-sectional designs. For instance, collecting repeated measurements of key variables can provide a more definitive evaluation of within-person change across time. Moreover, collecting repeated measurements can simultaneously increase statistical power for detecting changes while reducing the costs of conducting a study. In spite of the advantages over cross-sectional designs, repeated measures designs complicate the crucial process of selecting a sample size. Unlike studies with independent observations, repeated measurements taken from the same participant are correlated, and the correlations must be accounted for in calculating the appropriate sample size. Some current software packages used for sample size calculations are based on oversimplified assumptions about correlation patterns. As discussed later in the paper, oversimplified assumptions can give investigators false confidence in the chosen sample size. In addition, some current software may require programming skills that are beyond the resources available to many researchers.

In the present article, we describe methods for gathering the information required for selecting a sample size for studies with repeated measurements of normally distributed continuous responses. We also illustrate the process of sample size selection by working through an example with repeated measurements of pain memory, using the web-based power and sample size program GLIMMPSE.

## Tasks for selecting a sample size

### Select a data analysis method

For the sake of brevity, we will not elaborate on the fundamental question of choosing a data analysis method. Although statistical consulting will have value at any stage of research, the earlier stages of planning a study profit most from consulting. We assume the iterative process of choosing and refining the research goals, the primary outcomes, and the sampling plan has succeeded. In turn, we also assume that an appropriate analysis plan has been selected, which sets the stage for sample size selection.

### Select a power analysis method

One of the first steps in computing a sample size is to select a power analysis method that adequately aligns with the data analysis method [1]. As an example, consider a study in which a researcher plans to test whether veterans and non-veterans respond similarly to a drug. The researcher plans to control for both gender and age. The planned data analysis is an analysis of covariance (ANCOVA), with age as the covariate. In this case, a sample size calculation based on a two-group t-test would be inappropriate, since the planned data analysis is not a t-test. Misalignment between the design used for sample size calculations and the design used for data analysis can lead to a sample size that is either too large or too small [1], contributing to inconclusive findings.

In practice, mixed models have become the most popular method for analyzing repeated measures and longitudinal data. However, validated power and sample size methods exist only for a limited class of mixed models [2]. In addition, most of these methods are based on approximations, and make simple assumptions about the study design. In some cases, the planned data analysis has no published power analysis methods aligned with the data analysis. One possible method for finding reliable power or sample size when no power formulas are available is to conduct a computer simulation study. We recommend using appropriate software that has been tested and validated whenever it is available. Packaged software has the advantages of requiring less programming and less statistical sophistication.

Based on the current state of knowledge, we recommend using power methods developed for multivariate models to calculate sample size for studies using common mixed models for data analysis. For carefully built mixed models [3,4], power methods developed for multivariate models provide the best available power analysis. Technical background can be found in Muller et al. [1], Muller et al. [5], and Johnson et al. [6]. Another option is to use the large sample approximation for power described by Liu and Liang. They proposed a method to compute sample sizes for studies with correlated observations based on the generalized estimating equation (GEE1) approach [7].

### Model complex variance and correlation patterns

When planning a study with repeated measures, scientists must specify variance and correlation patterns among the repeated measurements. Failing to specify variance and correlation patterns aligned with the ones that will be seen in the proposed study can lead to incorrect power analysis [1].

The simplest variance pattern assumes equal variance among the repeated measurements. For example, measuring children’s mathematical achievement within a classroom makes it reasonable to assume equal variability across children, on average. In contrast, measuring mathematical achievement from the same children in grades 6, 7, and 8 could plausibly lead to increasing variability, decreasing variability, or stable variability. Variability in performance on a test of a certain skill could decrease across the grades due to a stable acquisition of the skill. On the other hand, variability of standardized test scores could remain unchanged due to careful test construction by the test developers. Repeated measurements of some variables may have any possible pattern of variance. For example, depending on the experimental condition, the metabolite concentrations in blood might increase, decrease, or remain unchanged across time.

Regarding correlation patterns, it is useful to think of them as having four types, in increasing complexity: (1) zero correlations (independent observations), (2) equal correlations, (3) rule-based patterns, and (4) unstructured correlations (no specific pattern).

The simplest model of correlations assumes a constant correlation, often referred to as an intra-class correlation, among all observations. If each observation records some aspect of a child’s performance within a classroom, then assuming a common correlation among any two children seems reasonable. In contrast, if the same child is measured in grades 6, 7, and 8, we expect the correlation between grades 6 and 8 to be lower than the correlation between grades 6 and 7. Correlations among the repeated measures from a single participant usually vary across time in a smooth and orderly fashion. Measurements taken close in time are usually more correlated than measurements taken farther apart in time.

Many rule-based patterns of correlation have been developed in the context of time series models. One common example of a rule-based pattern is the first-order autoregressive (AR1), a special case of the linear exponent first-order autoregressive (LEAR) family [8]. The AR1 and the more general LEAR patterns assume that correlations among repeated measures decline exponentially with time or distance. For example, in pain studies that examine the effects of interventions on patients’ memory of pain after treatment, the correlations among the measurements of pain memory from the same patient normally decrease over time. The relationship between memory of pain and passage of time can be modeled using the LEAR structure.

The unstructured correlation pattern assumes there are no particular correlation patterns among the repeated measures. Each correlation between any two repeated measurements may be unique. An unstructured correlation pattern requires knowing p × (p−1)/2 distinct correlations, with p being the number of repeated measures.

It is usually assumed that all participants in a study show the same pattern of correlation. Statistical methods are available to allow different correlation patterns among study participants. Although rarely used, a conscientious data analysis should include a meaningful evaluation of the validity of homogeneity of correlation pattern across groups of participants.

Each of the correlation patterns has limitations. Structured correlation patterns reflect special assumptions about the correlations among the repeated measures. The assumptions introduce the risk of choosing a pattern that is too simple, which can falsely inflate the type I error rate [3]. For example, the equal correlation pattern assumes that any pair of observations has the same correlation, no matter how far apart in time they fall. On the other hand, choosing an unstructured correlation pattern can be impractical because it requires estimating more parameters than the data support, which leads to a failure to converge. A flexible structure, such as the LEAR pattern, often provides the best compromise between too little complexity (equal correlation) and too much (unstructured correlation).

### Find valid inputs for sample size calculation

We illustrate how to find valid inputs for sample size calculations with an example drawn from a clinical study that used repeated measures of dental pain as the outcomes. The investigator plans to randomize the study participants to one of two groups, either control or treatment. Knowledge of the pain scale makes it reasonable to assume the data follow a normal distribution. The inputs needed to compute a sample size are (1) α, the Type I error rate, (2) the predictors implied by the design, (3) the target hypothesis being tested, (4) the difference in the pattern of means for which good power is being sought, (5) the variances of the response variables, and (6) the correlations among the response variables (Table 1). Finding the last three items in the list requires most of the effort.

**Table 1:** Inputs for power analysis for repeated measures design

**Source**	**Explanation**
Type I error rate (α)	The probability of claiming that an effect exists when in fact there is no effect; usually set at 0.01 or 0.05.
Predictor variables	The best set of predictors needs to be chosen; the categories of each predictor need to be specified.
Primary hypothesis	The primary hypothesis of interest needs to be specified. GUI power programs usually provide a list of possible hypotheses after all information is specified.
Smallest scientifically important difference	The minimum difference in the mean values of the response variable the investigators find important.
Variances of repeated measurements	Variance of each of the repeated measurements needs to be specified.
Correlations among repeated measurements	Correlations among pairs of the repeated measurements need to be specified.

Scientists designing a study usually know the Type I error rate, the predictors, and the target hypothesis. The type I error rate (α), chosen by the scientists, is the probability of claiming that an effect exists while in fact there is no effect (usually set at 0.01 or 0.05). Based on the scientific question at hand, scientists choose the best set of predictors and the target hypothesis to test.

Scientists must specify the smallest scientifically important difference. For the dental pain example, scientists must specify the minimum difference in the mean pain values they find important. The investigators designing the study need to make an educated choice for the mean difference of interest. For pain measured on a 0–5 continuous scale, a 0.5 change in pain level may not be clinically important, whereas a 1.0 change may be deemed important by the investigators.

Scientists must also specify the variance of each of the repeated measurements. Several strategies can be used to choose a variance value: (1) it may be estimated with data from previous studies, (2) it may be estimated with data from a pilot study, or (3) it may be an educated speculation based on experience. In the best case, it is possible to obtain a good estimate of a variance from a previous study. Researchers may have collected similar data on the response variable of interest in their previous studies. In other cases, published data on the response variable of interest may be available in the literature.

When using an estimate from a previous study, it is important to note that the variance needed for sample size calculation is the residual variance. The residual variance is the variance not explained by the predictors. An unadjusted variance of the response variable contains variation due to predictor variables included in the previous study. The same predictors may not be included in the study being planned. Suppose a new study will include a response variable from a relatively age-homogeneous population (e.g., college students at a large upper Midwest public university). The investigator needs to estimate a variance from a previous study in which the response variable was measured from people of all ages. Hence, only the residual variance can be used for sample size calculation, since the unadjusted variance of the response variable contains variability due to age.

Power analysis for repeated measures requires not just one but a set of variance values. As discussed earlier, the scientific context may provide a reasonable expectation for a pattern of change in variance. In practice, it is often possible to estimate one variance value based on data, and then specify the other variances based on the expected variance trend. Scientific context often provides a reasonable model. In general, growth, learning, and other developmental processes typically lead to monotonically decreasing variance, while aging, disease, and other patterns of degradation often create monotonically increasing variance.

Scientists must also specify the correlations among pairs of measurements. The same basic strategies for choosing a variance apply for choosing a correlation. It is often possible to estimate one correlation value based on data and then specify the other correlations based on the correlation pattern. When educated guesses are needed, a researcher’s experience and scientific restrictions guide the choice of a correlation. For example, behavioral scientists may expect to see survey reliabilities ranging from 0.25 to 0.75, while a biomedical engineer may expect instrument reliabilities of at least 0.90. As with variances, a chosen correlation must be a residual correlation, which is the correlation among the residuals for the repeated measures.

### Choose the right software

Many software packages and internet-based programs are available to perform sample size calculation for t-tests, a variety of ANOVA, and regression models. A small number of programs cover a limited range of repeated measures designs. Some of the programs are free and easy to install and use, but lack the ability to handle complex designs. For example, Java applets developed by Lenth (http://www.stat.uiowa.edu/~rlenth/Power/) provide power estimations for certain linear models such as t-tests, ANOVA, and linear regression studies [9]. Some programs are commercial products that can be used for a wide range of study designs, but can be prohibitively expensive and may also require great knowledge in statistical theories and strong computer programming skills. For example, POWERLIB is a free SAS/IML module that computes sample size and power for a wide variety of general linear univariate and multivariate models [6]. However, using this program requires a SAS software license, a strong knowledge of statistical theories, and SAS programming skills. Power Analysis and Sample Size (PASS, NCSS) computes sample size for a range of multivariate models (both linear and nonlinear), but it is a commercial product that needs to be purchased and installed on one’s computer. Some programs make simple statistical assumptions, and these assumptions limit their usefulness. For example, Optimal Design (OD) is a free sample size program with a graphical user interface (GUI) that allows users to compute sample size for longitudinal studies with multilevel designs [10]. However, the calculations for repeated measures use the over-simplifying assumptions of equal variances and correlations. In our experience, although variance may remain stable in longitudinal data in some cases, correlation never does.

Some programs from major software packages have built-in options for sample size calculation, but for univariate designs only. During the writing of the present manuscript, these programs include nQuery (nQuery Advisor, Statistical Solutions), SAS (GLMpower, SAS Institute Inc.), and SPSS (SamplePower, IBM Corporation). Given the continuing evolution of software, we urge the reader to be sure that the selected software has the abilities and features needed and meets professional standards for statistical methods and programming accuracy.

In general, software packages with a GUI interface are easier to use than those with a command line interface. However, one advantage of using command line interfaces is that they allow easy documentation and sharing of the whole power analysis process. Developed computer code can easily be shared with collaborators for review and re-used on other datasets. For reproducible research, it is good practice to document the whole power and data analysis process no matter what software package is used.

We recommend the program GLIMMPSE (URL: http://glimmpse.samplesizeshop.org/) for computing sample size for repeated measures and longitudinal designs. GLIMMPSE is a free, internet-based program that has two modes: Matrix Study Design Mode and Guided Study Design Mode. Matrix Study Design mode is designed for users with advanced statistical training and Guided Study Design mode is designed for applied researchers. GLIMMPSE requires no previous programming experience and provides a step-by-step, user-friendly interface to guide researchers through sample size and power calculations. In addition, GLIMMPSE allows saving the provided study design for future references. GLIMMPSE supports linear models with fixed predictor variables and linear models with fixed predictor variables plus one Gaussian covariate [11-13]. GLIMMPSE offers a host of variance and correlation patterns. The program has been extensively tested, with validation results available at the web site.

### Sample size calculation for a dental pain study

In this section, we use a real example to illustrate the process of gathering information for a sample size calculation for a repeated measures design. The Guided Study Design mode of GLIMMPSE is used for the calculation, but the investigator will need to follow the same steps to gather the required information, even if another program is preferred. Therefore, describing the technical details of GLIMMPSE is minimized. A manual on how to use GLIMMPSE is available at http://www.samplesizeshop.org.

### Overview of the pain study

In a previous study on therapeutic interventions for acute pain, Logan et al. discovered that an intervention instructing patients to pay attention only to the physical sensations in their mouth could greatly reduce sensory pain intensity during root canal therapy for patients who had both a high desire for control and low perceived control [14]. Since prior work shows that avoidance behavior builds as a result of the amount of pain recalled [15-18], the investigators are now planning to further examine the long-term effects of sensory focus on the perception of pain. A new set of participants will be recruited and tracked over time. The change in each participant’s memory of pain will be examined in the new study.

### Step 1: Specify the goal of the study

The effectiveness of therapeutic intervention for acute pain is often determined by measuring and comparing the amount of pain experienced and remembered by a patient. However, prior research has shown that short-term (hours to days) and long-term (months) memory of pain could be affected by different factors [18]. It has been argued that short-term memory of pain is an accurate reflection of the amount of pain experienced during the stimulus, since the recall and experience are temporally linked. On the other hand, long-term memory of pain may be influenced more by both temporal factors and cognitive and affective factors, many of which may be marginally related to the initial pain event. Therefore, for the new study, the primary goal proposed by the investigators is to determine if patients who are instructed to use a sensory focus have a different pattern of long-term memory of pain than patients who are not.

### Step 2: Specify the hypothesis

**Figure 1:**
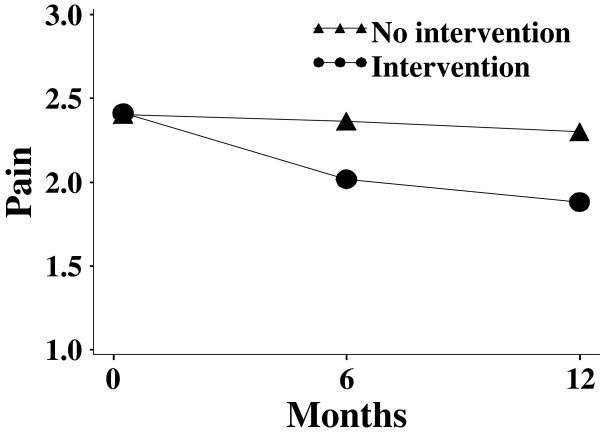
Hypothetical trends of pain memory.

With a repeated measures design we can test the main effect of intervention, by which the mean intervention effects averaged across the repeated measures are compared. We can also test trends across time. In this study, the investigators are interested in knowing if the trend of change is different between the intervention group and the non-intervention group. Therefore, the primary hypothesis of the study can be formally stated as a test of whether there is a time × intervention interaction. The hypothetical trends of pain memory for both groups are shown in Figure 1.

### Step 3: Specify the response variables

The main response variable of interest is memory of pain. It is a continuous variable that ranges from 0 to 5.0, with 0 meaning no pain remembered and 5.0 meaning maximum pain remembered [14]. Memory of pain will be assessed immediately after the dental procedure (Pain_0_), one week later (Pain_1_), six months later (Pain_2_), and twelve months later (Pain_3_). Pain_0_ will be measured in the clinic. Pain_1_, Pain_2_, and Pain_3_ will be measured through telephone interviews. The spacing in repeated measures is chosen based on the investigators’ knowledge of how pain memory changes over time.

### Step 4: Specify the predictor variables

The primary predictor of interest is the intervention (i.e., the audio instruction for participants to use a sensory focus during their respective dental procedures). In the new study, the Iowa Dental Control Index (IDCI) will be used to categorize and select patients [19]. Only patients with a high desire for control and low felt control will be recruited. Patients in this group will be selected and randomly assigned to either intervention or no intervention. Those in the intervention group will listen to automated audio instructions, in which they are told to pay close attention only to the physical sensations in their mouth [14]. Patients in the no-intervention group will listen to automated audio instructions on a neutral topic to control for media and attention effects. As in earlier studies, appropriate manipulation checks will be used [14].

### Step 5: Identify the variance and correlation patterns

Once the goals and the variables are specified, the next step is to specify the variance and correlation patterns among the repeated measures. In our case, the variance of difference between Pain_0_ was 0.96 in a previous study conducted by the investigators [14]. This variance of difference can be directly used as an estimate for the variances of the pain memory measures, Var(Pain_i_). As for the required correlations, 6 correlation values need to be estimated since there are 4 repeated measurements (Table 2). Prior research reports that the correlation between experienced pain and 1-week memory of pain is 0.60, and the correlation between experienced pain and 18-month memory of pain is 0.39 [18]. Therefore, it is reasonable to believe that the correlation between Pain_0_ and Pain_1_ is 0.60. In addition, since the investigators believe that the correlations decay smoothly across time, it is reasonable to assume the Pain_0_ - Pain_1_, Pain_0_ - Pain_2_, and Pain_0_ - Pain_3_ correlations are all larger than 0.39. Based on the trend of decay and the restrictive lower bound of 0.39, the investigators estimate that the Pain_0_ - Pain_1_, Pain_0_ - Pain_2_, and Pain_0_ - Pain_3_ correlations are approximately 0.6, 0.5, and 0.4, respectively (second column in Table 2). Following a similar thought process, the investigators estimate that the Pain_1_ - Pain_2_, Pain_1_ - Pain_3_, and Pain_2_ - Pain_3_ correlations are approximately 0.45, 0.40, and 0.45, respectively (Table 2).

**Table 2:** Estimated correlations among the pain memory measurements

	**Pain**_**0**_	**Pain**_**1**_	**Pain**_**2**_	**Pain**_**3**_
Pain_0_	-	-	-	-
Pain_1_	0.60	-	-	-
Pain_2_	0.50	0.45	-	-
Pain_3_	0.40	0.40	0.45	-

### Step 6: Generate a power curve and select an appropriate sample size

**Figure 2:**
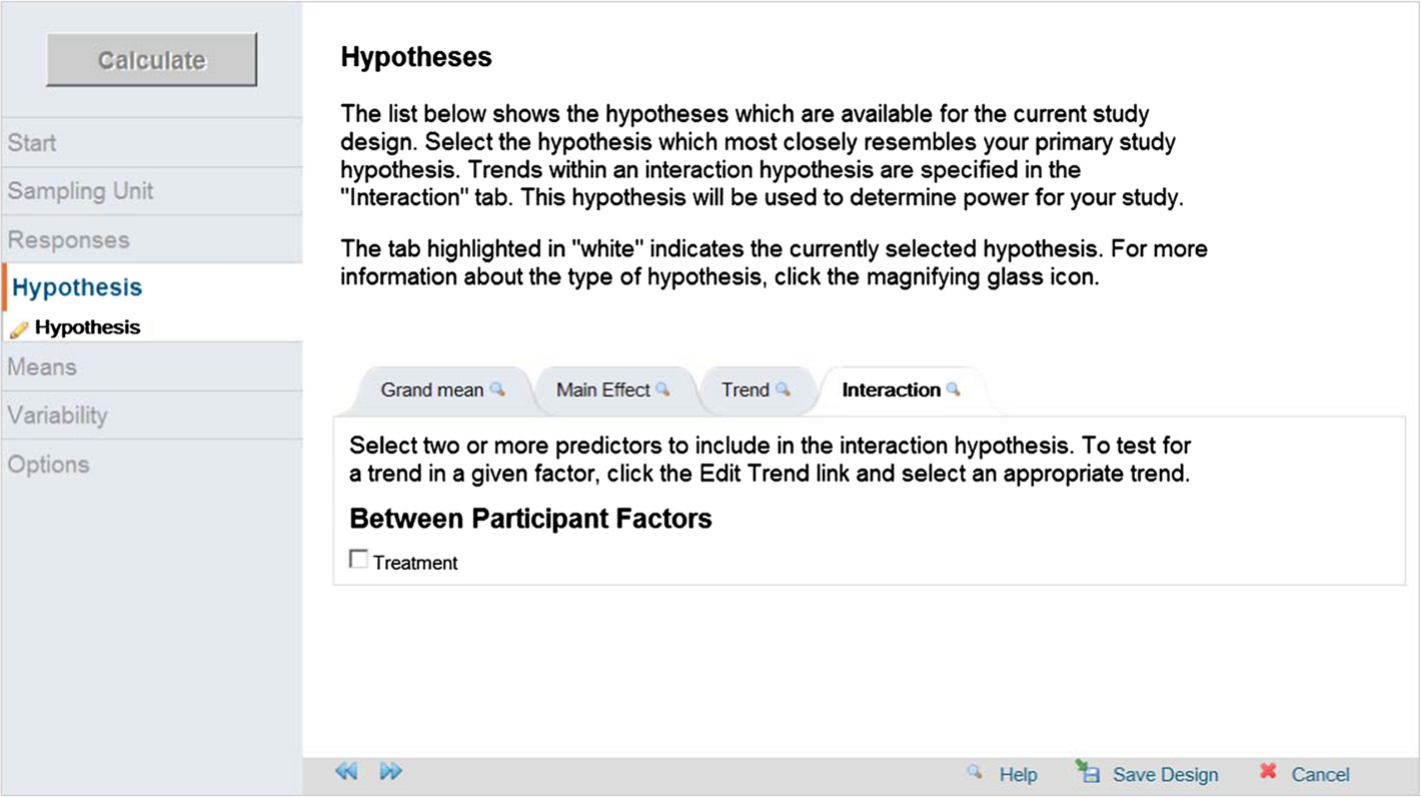
The hypotheses page in GLIMMPSE.

In GLIMMPSE, the user is prompted to enter the desired power values, type I error rates, study design variables, variances, and correlations. After these inputs have been made, GLIMMPSE will show a menu of possible hypotheses for the entered study design (Figure 2). For a repeated measures design, possible hypotheses include testing intervention main effect, trends across time, and time × intervention interaction. For our pain study, the hypothesis testing the interaction of time × intervention is chosen in GLIMMPSE.

**Figure 3:**
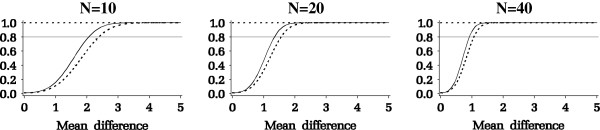
Power curves for the dental pain study.

Results from the power analysis are summarized in Figure 3. The y-axis is the power and the x-axis is the mean difference among the Pain_i_ measurements (e.g., Pain_2_ - Pain_1_). As seen in Figure 3, for a given desired power, the minimum detectable mean difference decreases as sample size increases. The investigators specified a minimal change in pain that they deem clinically important as a difference of 1.2 between the pain measures. A sample size of 40 patients per group, or a total of 80 patients, would give a power of at least 0.8 for testing the hypothesis of whether there is a time × intervention interaction.

## Additional practical considerations

Power analysis for studies with repeated measures can be complicated. It often involves solving a problem with many possible answers, such as specifying the variance and correlation patterns among the repeated measurements. Therefore, we recommend consulting with a statistician, if possible, when there are any unclear issues. The online sample size tool Glimmpse is designed such that it guides scientists through power analysis by asking questions about the study design. In the rest of this section, we provide additional practical advice on issues related to power analysis.

### Missing data

One limitation of the power analysis method based on general linear multivariate models is that it is a calculation for complete cases. In a complete case analysis, all repeated measurements on the same participant must be available. However, in longitudinal studies involving human participants, investigators often end up with missing data due to missed visits. One simple strategy to account for expected missing data is to propose an expected attrition rate, and then recruit more people accordingly. In our driving example, the investigators expect a maximum of 20% attrition for the one-year-long study, based on previous experiences with similar research projects. Therefore, they need to collect data for 20% more participants in order to achieve the desired power. On the other hand, this method does not consider partial information that might be obtained from participants with missing visits or dropout participants. There are sample size methods developed for these scenarios, but discussing them is out of the scope of this article [20]. Furthermore, as always, the possibility of non-randomly missing data must be carefully examined by checking the study design and data collection procedures once the data have been collected.

### Power for more than one primary hypothesis

Due to cost and ethical issues, scientists often want to test more than one hypothesis in a study. Each power analysis must be based on one specific hypothesis using a pre-planned data analysis method. With a modest number of primary analyses, a simple Bonferroni correction is typically applied to help control the Type I error rate. For example, with 4 primary hypotheses, a Type I error rate of *α* = 0.05 ÷ 4 = 0.0125 would be used. Having conducted 4 power analyses leads to 4 different power values or ideal sample sizes. In the absence of time, cost, and ethical concerns, the scientist may choose the largest sample size to guarantee power for all 4 tests.

### Covariates

In addition to categorical variables, continuous variables are sometimes included in studies as predictors or baseline covariates. A baseline covariate is the first measurement (before treatment) of a continuous response measured repeatedly over time. It is included as a predictor variable to control for differences in the starting values of the response. Using a baseline covariate that controls a large proportion of the variance of the response increases the statistical power of the data analysis, but also complicates the calculation of sample size. However, current knowledge of the closed form approximation covers only sample size and power calculation in general linear multivariate models with a single, continuous, normally distributed predictor variable [13]. Glueck and Muller reviewed the limited approximate power methods that are available for adjusting for covariates [13].

## Conclusions

Using a repeated measures design improves efficiency and allows testing a time × treatment interaction. In practice, the critical task of selecting a sample size for studies with repeated measures can be daunting. In this article, we described a practical method for selecting a sample size for repeated measures designs and provided an example. In addition, we gave practical advice for addressing potential problems and complications.

## Competing interests

In previous years, Dr. Muller has briefly served as a paid consultant to SAS Institute.

## Authors’ contributions

YG and KM were responsible for the conception and writing of the manuscript. HL designed the example dental study and assisted with writing. DG assisted with the conception and writing. All authors read and approved the final manuscript.

## Pre-publication history

The pre-publication history for this paper can be accessed here:

http://www.biomedcentral.com/1471-2288/13/100/prepub
